# The Associations between Body Mass Index of Seven- and Eight-Year-Old Children, Dietary Behaviour and Nutrition-Related Parenting Practices

**DOI:** 10.3390/medicina55010024

**Published:** 2019-01-21

**Authors:** Justina Vaitkevičiūtė, Aušra Petrauskienė

**Affiliations:** Health Research Institute, Faculty of Public Health, Medical Academy, Lithuanian University of Health Sciences, Tilžės str. 18, LT–47181 Kaunas, Lithuania; ausra.petrauskiene@lsmuni.lt

**Keywords:** children, dietary behaviour, body mass index, nutrition-related parenting practices, family

## Abstract

*Background and objective*: Body mass index (BMI) is one of the key indicators used to measure the growth of children. It could be affected by the children’s nutrition, which is essential for the proper development of the child. Nutrition of children could be affected by many environmental factors, for example, the socioeconomic environment of the family. The aim of this study was to identify the associations between the BMI of seven- and eight-year-old children, dietary behaviour and nutrition-related parenting practices. *Materials and Methods:* The study was carried out as part of the World Health Organization European Childhood Obesity Surveillance Initiative (WHO COSI). Data were collected using two instruments: objective anthropometric measurements and a questionnaire. The target participant group was 3969 Lithuanian first-formers. Factor analysis was used to summarise questions from the family form. Linear regression analysis was used to identify the associations between various factors and the BMI value of the children. The association between two groups of factors was analysed using Spearman correlation. *Results:* Factors of dietary behaviour like unhealthy food and proteins were significantly positively associated with BMI in children, while consumption of plant-based, dairy and confectionery items was significantly negatively associated with BMI. Factors of nutrition-related parenting practices like control of unhealthy food, food as a reward or punishment, and mealtime were significantly positively associated with BMI, while encouragement, pressure to eat, and liberal attitude were significantly negatively associated with BMI. The strongest associations were between control of unhealthy food and unhealthy food; cost of and preferences for food and plant-based food; variety of food and proteins; variety of food and plant-based food compared to other associations. *Conclusions:* The dietary behaviour and nutrition-related parenting practices were associated with BMI in children.

## 1. Introduction

Overweight and obesity among children is one of the greatest public health challenges of the 21st century. According to World Health Organization European Childhood Obesity Surveillance Initiative (WHO COSI) data, this problem is still particularly relevant in South European countries such as Spain, Italy, Greece and Cyprus. However, a significant decline in the prevalence of elevated body weight among children was observed in Greece, Italy, Portugal, and Slovenia. Growing trends were observed in Latvia, Lithuania and Bulgaria [1]. Similar results were determined by another important adolescent study—Health Behaviour in School-Aged Children (HBSC), wherein excess body weight issues were observed in Malta, Greece, and Italy [2]. It is also important to mention that the prevalence of underweight children is also an issue. This problem is relevant not only in poorer developing countries but also in developed Western countries such as Spain where, in the last decade, increasing numbers of underweight children were observed [3,4]. Therefore, programs for children should be directed towards promoting a healthy lifestyle for all sizes, independently of the BMI of children [3,5].

Nutrition is identified as one of the essential factors related to the weight of children. In evaluations of the relationship between food and the BMI, most common associations were found between overweight, obesity and more frequent intake of unhealthy, fatty food and soft drinks [6,7,8]. According to Beckerman, food choices were shaped in early childhood, but they were also dependent on the environment children live in (policies, culture, media, community, various programs, etc.) [9]. One of the factors influencing the BMI of children was nutrition-related parental practices: the way parents develop their child’s eating habits [10]. Gevers has highlighted the most important nutritional parenting practices, such as modelling of eating behaviour, limiting certain types of food, the pressure to eat, food as a reward, food availability and choices available at home [5]. Systematic reviews identified that an uninvolved, highly protective or strict parenting style was associated with higher BMI in children, while authoritative parenting was associated with healthy BMI and pressure to eat was associated with a lower BMI [10,11].

Lithuania has been participating in the WHO COSI survey since 2008. The main aim of the study was to monitor the growth of primary school age children and to evaluate their school and family environment. According to the results of this study, in 2016 in Lithuania, the prevalence of overweight, including obesity, among seven- and eight-year-old children was about 20%. In addition, this indicator increased significantly in comparison with 2008. At the same time, a slight increase in the prevalence of underweight children was observed, and in 2016 it was about 10% [4], while the proportion of children at normal weight decreased with each year of the survey. A few studies analysing the associations between body weight and sedentary behaviour, consumption of junk food, and school environment in Lithuania were performed [12,13,14], but no studies were found assessing the association between BMI of children and family environment. Therefore, taking into account similar studies carried out in other countries that evaluated the association between the family environment and the BMI of children, it is important to evaluate the associations that are specific to Lithuania, how much they coincide with the data of other studies and how different the family eating culture is [10,15,16]. The family is the most important environment for a child and has an impact on what, how, how much, how often, where and with whom the children eat, and what habits they will develop in the future. Therefore, the aim of this study was to identify the associations between the BMI of seven- and eight-year-old children, dietary behaviour and nutrition-related parenting practices.

## 2. Materials and Methods

### 2.1. Study Design and Subjects

This study was carried out as part of the COSI study and was based on an international protocol approved by the WHO. The COSI aim is to measure children’s weight and height in order to follow trends in overweight and obesity in children and monitor the progress of different parameters to allow inter-country comparisons. COSI protocol included the option to gather information through a COSI Family Form [17].

In this study, data were collected using two instruments: objective anthropometric measurements and a questionnaire—the COSI Family Form. Specifically, extra questions related to nutrition-related parenting practices were developed and added to the Family Form.

The data were collected during a cross-sectional survey conducted in 2016 (April–May) in Lithuania. The target group was first-formers (7–8-year-olds). The representative sample of Lithuanian first-formers was based on the number of target age group children in each county of Lithuania (in proportion to each county, according to the data of the Lithuanian Department of Statistics). Schools were selected randomly from the list of schools received from the Ministry of Education and Science. One hundred two schools from all 10 counties of Lithuania agreed to participate in this study. All first classes in the selected schools and all children registered in the class were invited to participate in the survey—5537 children in total. Data from 3969 participants were analysed.

### 2.2. Research Ethics

Envelopes with Informed Consent Form and Family Form were distributed and handed over to parents with the help of teachers during the first visit to schools. During the next visit, envelopes were collected. Measurements were performed on those children who participated in their classes on the day of the second visit to the school and written informed consent for the child’s participation in the survey was obtained from both parents. The research protocol was approved by the Lithuanian Bioethics Committee (approval date 13 March 2008 No. 16 and its extension 9 January 2013 No. 6B–13–17). The study was carried out following the rules of the Declaration of Helsinki.

### 2.3. Anthropometric Measurements (Child’s Record Form)

Anthropometric measurements (weight and height) were performed according to COSI protocols [18,19] by trained and standardized examiners. Child consent was always obtained before the measurements. The following data were collected using a Child’s Record Form (filled out by the examiner): name, surname, sex, date of birth, date of measurement, the reason given by a child who did not give permission to be measured, and records of measured weight and height. Children were asked to take off their shoes, heavy clothes and items such as wallet, keys, mobile phone, hair ornaments or braids, etc. The clothes worn by a child while being measured were noted in Child Record Form selecting one of the predefined types of clothing: “Underwear only,” “Gym clothes (e.g., shorts and t-shirt only),” “Light clothing (e.g., t-shirt, cotton trousers or skirt),” “Heavy clothing (e.g., sweater and jeans)” or “Other (please specify).” During data analyses, body weight was adjusted for the weight of the clothes worn by the children when they were measured.

The same anthropometric equipment was used in the measurements at all schools. Measurements were carried out by the SECA’s Portable Medical Scales and Height Boards. Body weight was measured in kilograms and recorded to the nearest 100 g (0.1 kg) unit. Height was measured in centimetres and the reading taken to the last completed 1 mm (0.1 cm).

BMI was calculated using the formula: weight (kg) divided by height squared (m^2^).

### 2.4. Questionnaire (Family Form)

Dietary behaviour and nutrition-related parenting practices were evaluated using the Family Form. Parents were asked, “In a typical week, how often does your child eat or drink the following kinds of food or beverages?” There were five predefined options for the answer: Never, Less than once a week, Some days (1–3 days), Most days (4–6 days) and Every day. The second part of the questionnaire aimed to evaluate the nutrition-related parenting practices assumed to be related to children’s nutritional status. A literature review was performed and 34 items to evaluate nutrition-related parenting practices were developed (Table 1). Items have been published elsewhere [20,21,22,23] and in the present study versions of statements were modified. A five-point Likert response scale (ranging from “Totally disagree” to “Totally agree,” with a neutral midpoint “Neither”) was used to evaluate the level of agreement with nutrition-related parenting practices items.

### 2.5. Data Analysis

Factor analysis and Varimax rotation were used to summarise questions in the Family Form and to create new variables—factors that were considered to load on a factor if factor loading was >0.4. Items with one or more high loadings were included in the factor where its loading was the highest. Dietary behaviour was reduced from 20 items to 5 factors (Table 2) and explained 50.1% of the total variance. Two items were eliminated from the analysis due to factor loading <0.4. The Kaiser‒Meyer‒Olkin (KMO) Test of Sampling Adequacy for the first factor analysis was 0.751. Cronbach’s alphas were calculated. Values ranged from 0.632 to 0.72 for scales of dietary behaviour. Nutrition-related parenting practices were reduced from 34 items to 11 factors (Table 1) and explained 62.44% of the total variance. Two items were eliminated from the analysis due to factor loading <0.4. Kaiser‒Meyer‒Olkin (KMO) test of sampling adequacy for the second factor analysis was 0.803. Calculated values of Cronbach’s alpha ranged from 0.615 to 0.829 for scales of nutrition-related parenting practices.

Medians, 25th and 75th percentiles (P) were used for the description of the factors (Table 1 and Table 2). The linear regression analyses were undertaken to examine the associations between factors of dietary behaviour, nutrition-related parenting practices and BMI of children. Associations between two groups of factors (dietary behaviour and nutrition-related parenting practices) were evaluated by bivariate correlation analyses and Spearman correlation analysis. All *p*-values <0.05 were considered statistically significant. Statistical analyses were performed by IBM SPSS Statistics, version 20.0 for Windows.

## 3. Results

The total sample size included 3969 children, and 3844 of them were measured anthropometrically. Just over half of the children were boys (50.9%). The mean age was 7.33 (SD (standard deviation): 0.47). Most of the children were of normal weight (70.1%), and every fifth first-former was overweight (including obesity) (19.8%).

Table 3 shows the results of linear regression analysis between dietary behaviour and BMI of children. Consumption of unhealthy food and proteins was significantly positively associated with BMI, showing a relationship between higher BMI in children and more frequent consumption of the mentioned foods. In contrast, more frequent consumption of plant-based, dairy and confectionery food groups were significantly negatively associated with BMI in children. For instance, every unit increase in the plant-based factor was associated with a decrease in predicted BMI of 0.127 kg/m^2^.

Associations between nutrition-related parenting practices and BMI in children are presented in Table 4. Control of unhealthy food, food as a reward or punishment and mealtime were significantly positively associated with BMI in children, suggesting that more parental control over the consumption of unhealthy food, food being used as a reward or punishment and a lower importance of mealtime to the family were related with increased BMI.

Stronger agreement with items related to encouragement, pressure to eat and liberal attitude was significantly negatively associated with BMI in children.

Correlations between established factors of eating habits and nutrition-related parenting practices were detected (Table 5). However, the correlation coefficients were weak. Frequent parental control of children’s eating was correlated with consumption of less unhealthy food (−0.255). A negative correlation was found between cost of food and family preferences of food and consumption of fruits and vegetables (−0.278) by children. The importance of the variety of food was positively correlated with proteins like meat (0.222), fruits and vegetables (0.324).

## 4. Discussion

During a survey of children’s nutrition among the seven- and eight-year-olds in Lithuania, it was found that the increased intake of unhealthy and more protein-rich food was associated with higher BMI in children and, conversely, more frequent use of plant-based and dairy products was associated with lower BMI. An unexpected result was found when assessing the interactions with sweet food use—lower BMIs were associated with the more frequent consumption of confectionery. In assessing nutrition-related parenting practices, we found that increased control of unhealthy food consumption, the use of food as a reward or punishment, and the lower importance of family mealtime were significantly associated with higher BMI in children. Encouragement, pressure to eat and a liberal attitude to nutrition were associated with lower BMI in children.

In addition, associations between dietary behaviour and nutrition-related parenting practices were identified. Parents’ control of unhealthy food was correlated negatively with consumption of less unhealthy food. Furthermore, a negative correlation was found between cost of food and family preferences of food and children’s consumption of fruits and vegetables. A positive correlation was found between the importance of a variety of food and consumption of fruits, vegetables and proteins like meat.

Various studies in other countries have also found similar results and identified associations between the frequencies of consumption of unhealthy, high-fat food [6,7,8], soft drinks [8] and higher BMI in children. In the Brazilian study, several products were identified as obesogenic: sweets and sugar, typical Brazilian dishes, pastries, fast food, oils, milk, cereals, cakes and sauces [24]. Compared to normal-weight participants, overweight/obese participants had higher energy and macronutrient intake and consumed more cereal grains, meat/fish, flavoured milk, and soft drinks during main meals, and street-side snacks and confectionery between meals [25]. Wall found an inverse association between BMI and higher consumption of fruit, vegetables, pulses and nuts in adolescents [26]. Therefore, the results of the mentioned studies partly confirmed the results of our study, though we have found an opposite and unexpected relationship between the intake of sweet food and lower BMI in children, i.e., the consumption of sweet products such as chocolate, sweets and confectionery was more frequent among children of lower weight. Our study is not the only one that has not detected a link between higher BMI in children and the consumption of sweet food [27]. Children naturally prefer higher levels of sweet and salty tastes [9,28]. Previous studies have shown that children who were picky eaters were more likely to be of lower weight [23,29,30]. Therefore, we can assume that picky eaters may be more likely to choose sweet foods, while parents are less likely to regulate the intake of that sweet food. Another explanation could be that larger portions of sweet food are consumed by physically active children who burn calories through active play or exercise. Supposedly parents of underweight or normal-weight children have a more liberal attitude to the use of sweets, as their children do not have problems associated with excessive body weight. Meanwhile, the parents of children with higher weight paid more attention to the nutrition of children and restricted the use of confectionery more frequently. However, one of the most rational explanations is that the results obtained could be as a limitation of cross-sectional survey.

As mentioned previously, our study identified a positive association between unhealthy food consumption, control of unhealthy food intake and higher BMI in children. Warkentin also observed a positive association between parental restriction and BMI z-scores of children [31]. Other authors have also found similar results [10,32,33,34,35]. Parental control could be defined as a restriction, the pressure to eat, and the use of food items as a reward. According to some scientists, control may have had the opposite impact on eating behaviour to what is expected [36]. Two types of control could be distinguished: “overt control” (the child is told when, how much, etc. s/he should eat) and “covert control” (when parents create conditions for healthy eating) [37,38]. “Overt control” is more beneficial for the formation of a child’s dietary behaviours than “covert control,” which seems to highlight the problem of excessive weight.

Scientists pointed out that various restrictions could be effective only temporarily. For example, restricting the intake of candy, unhealthy or fatty food for a longer period of time may have the opposite effect, i.e., the child will prefer foods high in fat and sugar [39,40]. KOALA’s study has found that a higher BMI z-score in five-year-old children was associated with more frequent dietary restrictions and the promotion of healthy foods [23,37]. However, evaluating BMI changes in children from five to seven years old, it was found that their BMI z-score declined due to an encouragement to eat healthier. Higher parental control had no impact on changes in the BMI z-score of children [23,37]. On the contrary, other studies have determined that the BMI of children with more frequent parental control was higher [41]. Nevertheless, there were studies showing that pressure to eat and “overt control” were involved in the reduction of BMI in children [37]. It was observed that parents of obese children had higher rates of discouragement of desserts compared to parents of normal-weight children; however, it has been identified that due to restrictions the consumption of vegetables has decreased 3-fold, and the consumption of unfamiliar vegetables was even lower [42]. Promoting eating healthy foods and monitoring whether the child is overeating unhealthy foods has been associated with higher fibre intake and less common use of added sugars [23]. Myers has reported that participation in educational programmes such as meal planning, encouragement to eat homemade food and choosing healthier snacks has been effective, and it was observed that in involved families the BMI of children decreased [43].

Paradoxically, parents were more likely to apply dietary restrictions to children with higher body weight as well as use certain foods as a reward or punishment. Other authors claimed that a greater risk of obesity was for those children whose parents used candy as a reward for good behaviour [44,45]. Children preferred food that was used as a reward [39]. Moreover, it was found that when children were raised in this way they used fast food and soft drinks more often and thus shaped their eating habits improperly [46].

In our study, an association was found between lower BMI in children and more frequent encouragement and pressure to eat by parents. Other studies have also confirmed that parents used pressure to eat more often for children of lower weight [29,32,33,34,37,47]. The findings of the systematic reviews showed that an uninvolved, indulgent or highly protective/strict parenting style was associated with higher BMI, authoritative parenting with healthy BMI, and pressure to eat with a lower BMI in children [10,11]. It was determined that picky eaters were encouraged to eat healthy less often; however, these children also experienced greater parental control [23]. In addition, picky eaters tended to be of lower weight [29,30]. We could assume that parents are more concerned about how much their child is eating and less concerned about the quality of food. Contrary results were found in the Netherlands, where it was identified that parents of picky eaters restricted the consumption of unhealthy food more frequently, compared to parents of non-picky eaters, while parents of non-picky eaters encouraged children to eat healthy food more often [29]. Children with a picky eating style had a lower risk of being overweight in the future compared to normal-weight children, but their energy intake only slightly differed if compared with non-picky children [29]. Therefore picky eating, although it raises parental concerns and pressure to eat, is not such a big issue. It is only important that the child get enough nutrients and microelements needed for proper growth and development.

Overall, we can conclude that scientists presented controversial results on the impact of parental practices on the BMI of children. Increased parental control may be the result or the cause of increased BMI in children. Parents may be involved in the control of the diet of their children in order to reduce their weight. However, another hypothesis could be that greater parental control, lack of self-control in children and lack of parental competence could be the reasons for the increasing weight of the child. In addition, food control can have a negative impact on how much a child eats—both healthy and unhealthy foods. Parents should avoid their children following restrictive diets, which can lead to a lack of certain nutrients for a growing child.

Wansink has found that BMI was lower in those children who were eating at the dining table in the kitchen or dining room [48], while the BMI of children who were eating in other rooms (living room or bedroom) was higher [49]. Lee has found an association between higher frequency of eating with the family and preferred eating habits, but the association between frequency of eating with the family and the consumption of unhealthy food was not determined [50]. A 10-year cohort study has found that adolescents who never ate with the family had a higher chance of being overweight or obese compared to those who had meals with the family [51]. Meanwhile, Berge, while studying preschool children, has not established an association between eating together and BMI in children [52]. Other authors stated that eating together was more related to the quality of food but not to body weight [53]. More frequent eating at home was associated with increased consumption of vegetables, fruits, fish, farinaceous, dairy products and eggs and lower amounts of meat, sweets and soft drinks [54]. Decreases in energy received from non-homemade food were associated with lower BMI and fat-free mass in children, as well as a higher quality of nutrition (increased intake of fibre, fruits and vegetables; higher amount of energy from carbohydrates; and decreased total energy from added sugars, fats and soft drinks) [55]. It is possible that children, while eating together with their families, learned from their parents and took on their diet; furthermore, parents could use “covert” or “overt” control. Our study has also identified that the BMI of children was higher when the mealtime was less important to the family. We assume that the importance of eating together as a family affects physical health and mental health, because the family eats as well as communicates. This shows how important it is for family members to interact and communicate with each other. After all, in a variety of cultures, including Lithuania, the importance of and attention to a person is shown by providing food, yet this kind of behaviour does not always have a positive effect on health. According to the research data, in families where grandparents helped to take care of children, the risk of overweight and obesity of children was higher [56].

Attention should be paid to the quality of food served at home. Food served at home is more likely to be made at home, healthier and of better quality compared to non-homemade. However, it is dependent on the cultural context. There are countries where families eat together often, but they do not eat at home or their food is not homemade. This may affect the quality of food and the weight of children as well. In Lithuania, frequent visits to fast food restaurants are not very common compared to other Western countries. Historical circumstances have led to the late emergence of fast food restaurants in Lithuania, and for a large proportion of the population visits to these restaurants are still a luxury, unlike in the United States, where the people frequenting fast food restaurants most often are of lower socioeconomic status. However, tendencies that are typical of Western countries are also observed in Lithuania, when fewer everyday meals are being prepared at home. Traditionally, Lithuanian homemade food is higher in fat compared to the cuisines of other regions. Therefore, in assessing the family environment, it is important to consider the cultural context. Moreover, it is important to observe the variety. It is important to mention that the cultural environment is changing; society is increasingly talking about the impact of nutrition and physical activity on health, while some people are choosing to use homemade food. We hope that these tendencies will increase and in the future will contribute to healthy child eating habits.

The problems of overweight or underweight in children should be noticed and effective actions implemented. Parenting practices that empower children to self-regulate energy intake, increase the availability of nutritious and healthy products at home and model healthy choices are important for children’s weight and diet quality [32]. Cooking and growing vegetables at home could be a way to encourage children to get acquainted with food, its origins and how to love it. It is important that parents set an example. A healthy and balanced diet is essential for all children, regardless of their BMI.

### Limitations and Strengths

One of the limitations of this study was that the dietary behaviour and nutrition-related parenting practices were evaluated in a cross-sectional study, so it was not possible to determine whether parenting was the cause or the consequence of higher BMI in children. In addition, other characteristics of the child’s personality or character could have an impact on eating habits. Furthermore, children’s response to parental control depends on the child’s temperament [28]. The nutrition of children depends on parenting styles as well as on the general environment, parenting and family climate, and culture. During this study, only one element—nutrition-related parenting practices—was evaluated. Furthermore, the questionnaire, which was designed to assess the family environment, was filled in by the parents themselves, so answers may be less reliable due to the bias of respondents.

Despite the limitations, this study analysed an area that is not adequately investigated in Lithuania—the association between nutrition-related parenting practices and BMI in younger school-aged children. Objective anthropometric measurements of the representative sample of seven- and eight-year-old children were performed using standardized methodology. Due to historical circumstances, Lithuanian residents still differ in their dietary habits from those in Western European countries, but similar trends in overweight and obesity issues are becoming more and more relevant.

The aim of our study was to draw attention to family nutrition factors affecting BMI in general, not only excess body weight, but also the problem of underweight, which is less discussed not only in Lithuania, but in other countries as well. According to Lithuanian Health Behaviour in School-Aged Children (HBSC) 2002 [57] and 2006 [58] study results, the prevalence of overweight including obesity was the lowest among all participating countries. The Lithuanian COSI [4] data showed trends of increasing rates of excess body weight and presence of underweight. These problems are growing, but they are still insufficiently explored and not always recognized. In Lithuania we have had regulations on children’s nutrition in schools since 2010; these regulations are becoming stricter over the years, but despite the efforts of policy makers, the situation is still not improving. Since parents are responsible and have the greatest influence on the diet of children in their early years, it is very important to determine how nutrition-related parenting practices are linked to physical development in children.

## 5. Conclusions

This study has identified that the consumption of unhealthy food and proteins was positively associated with BMI in children, while plant-based, dairy and confectionery food groups were associated negatively. Control of unhealthy food, food as a reward or punishment and mealtime were positively associated with BMI and encouragement/pressure to eat and liberal attitude were negatively associated with BMI.

## Figures and Tables

**Table 1:** Items and factor loadings for the scales of nutrition-related parenting practices reported in the questionnaire completed by the parents of seven- to eight-year-old children.

Factors	Items	Factor Loading	Median	25th P	75th P
**Factor 1**	**Control of Unhealthy Food**		0.006	−0.463	0.773
8.38% variance	I have to ensure that my child does not eat too many sweets	0.851			
	I have to ensure that my child does not eat too many unhealthy snacks and fast food (potato chips, peanuts, pizza)	0.847			
	I have to ensure that my child does not drink too many soft drinks	0.835			
	I am hiding some food products from my child	0.410			
**Factor 2**	**Cost of and Preferences for Food**		−0.048	−0.734	0.525
7.65% variance	I do not buy many fruits because they cost too much	0.840			
	I do not buy many vegetables because they cost too much	0.829			
	I do not buy many fruits because my family do not like them	0.720			
	I do not buy many vegetables because my family do not like them	0.707			
**Factor 3**	**Taking Care of Family Food**		0.021	−0.595	0.636
6.54% variance	I enjoy cooking for the family	0.741			
	I like trying new recipes	0.657			
	I usually plan what we will eat the next day	0.562			
**Factor 4**	**Encouragement**		−0.110	−0.563	0.721
5.99% variance	I encourage my child to try new foods	0.750			
	I encourage my child to eat healthy foods before unhealthy ones	0.736			
	I encourage my child to eat a variety of foods	0.705			
**Factor 5**	**Pressure to Eat**		−0.007	−0.728	0.748
5.59% variance	If my child says, ‘‘I’m not hungry,’’ I try to get her/him to eat anyway.	0.831			
	If I did not guide or regulate my child’s eating s/he would eat much less than s/he should	0.813			
	My child should eat all of the food on her/his plate.	0.658			
**Factor 6**	**Preparation of Food**		0.048	−0.618	0.660
*5.27*% variance	When cooking food, I usually choose something stewed, steamed or oven-cooked	0.706			
	In my family, food is more often baked in a pan than steamed or cooked in the oven	−0.632			
	When I make food, I am choosing healthier foods	0.524			
	I model healthy eating for my child by eating healthy food myself	0.488			
**Factor 7**	**Food as a Reward or Punishment**		−0.075	−0.675	0.640
*5.24*% variance	I use food as a reward for good behaviour	0.783			
	I use food as a punishment for bad behaviour by not giving chips, sweets, ice cream, etc.	0.769			
**Factor 8**	**Variety of Food**		0.085	−0.524	0.689
*4.79*% variance	My child eats a variety of foods	0.851			
	My child eats plenty of vegetables and fruits	0.826			
**Factor 9**	**Liberal Attitude**		0.025	−0.667	0.642
*4.56*% variance	I allow my child to eat snacks whenever s/he wants	0.739			
	I let my child eat whatever s/he wants	0.572			
	I allow my child to leave the table, even if the family is not done eating	0.507			
**Factor 10**	**Childs Involvement**		0.053	−0.581	0.654
*4.40*% variance	I involve my child in planning family meals	0.779			
	I allow my child to help prepare family meals	0.754			
**Factor 11**	**Mealtime**		−0.035	−0.764	0.678
*4.03*% variance	In our family, it is often difficult to find a time when family members can sit down to a meal together	0.755			
	It is important that our family eat at least one meal a day together	−0.699			

P—Percentiles; *n* = 3006; Kaiser‒Meyer‒Olkin Test (KMO) of sampling adequacy—0.803; total variance explained 62.44%; 5-point Likert response scale (ranging from “Totally disagree” to “Totally agree,” with a neutral midpoint “Neither”) was used.

**Table 2:** Items and factor loadings for the scales of dietary behaviour reported in the questionnaire completed by the parents of seven- to eight-year-old children.

Factors	Items	Factor Loading	Median	25th P	75th P
**Factor 1**	**Unhealthy Food**		−0.055	−0.646	0.461
13.28% variance	Soft drinks	0.755			
	Diet or “light” soft drinks	0.728			
	Savoury snacks like potato chips, corn chips, popcorn or peanuts	0.711			
	Foods like pizza, French fries, fried potatoes hamburger, sausage or meat pies	0.598			
	Flavoured milk	0.532			
**Factor 2**	**Proteins**		0.034	−0.609	0.672
11.06% variance	Meat (any kind of meat and meat products)	0.816			
	Red meat	0.735			
	Chicken	0.634			
	Products of meat	0.450			
**Factor 3**	**Plant-Based**		0.009	−0.690	0.685
9.49% variance	Fresh fruits	0.758			
	Vegetables (fresh and boiled)	0.714			
	Cereal or flakes	0.523			
	100% fruit juice	0.458			
**Factor 4**	**Dairy**		−0.038	−0.697	0.644
8.21% variance	Yoghurt, milk pudding, cream cheese/quark or other dairy products	0.640			
	Whole fat milk	0.639			
	Cheese	0.632			
**Factor 5**	**Confectionery**		−0.057	−0.716	0.583
8.06% variance	Sweet treats like a candy bar or chocolate	0.820			
	Foods like biscuits, cake, doughnuts or pie	0.783			

P—Percentiles; *n* = 3169; Kaiser‒Meyer‒Olkin Test (KMO) of sampling adequacy—0.751; total variance explained 50.10%; five predefined options of answer used: Never, Less than once a week, Some days (1–3 days), Most days (4–6 days) and Every day.

**Table 3:** Linear regression coefficients and 95% CI for the association between factors of dietary behaviour and BMI of seven- to eight-year-old children.

Dietary Behaviour	BMI (kg/m^2^)		
	Unstandardized Regression Coefficient (β) (95%CI)	Standardized Regression Coefficients (β)	*p*
Unhealthy Food	0.152 (0.063 to 0.241)	0.060	0.001 *
Proteins	0.149 (0.059 to 0.238)	0.059	0.001 *
Plant-Based	−0.127 (−0.217 to −0.037)	−0.050	0.006 *
Dairy	−0.112 (−0.201 to −0.022)	−0.044	0.014 *
Confectionery	−0.175 (−0.265 to −0.085)	−0.069	<0.001 *

BMI—body mass index; CI—confidence interval; * *p* < 0.05.

**Table 4:** Linear regression coefficients and 95% CI for the association between factors of nutrition-related parenting practices and BMI of seven- and eight-year-old children.

Nutrition-Related Parenting Practices	BMI (kg/m^2^)		
	Unstandardized Regression Coefficient (β) (95% CI)	Standardized Regression Coefficients (β)	*p*
Control of Unhealthy Food	0.132 (0.046 to 0.218)	0.053	0.003 *
Cost of and Preferences for food	0.073 (−0.014 to 0.159)	0.029	0.099
Taking Care of Family Food	0.027 (−0.059 to 0.114)	0.011	0.535
Encouragement	−0.110 (−0.196 to −0.024)	−0.044	0.012 *
Pressure to Eat	−0.704 (−0.790 to −0.618)	−0.282	<0.001 *
Preparation of Food	−0.063 (−0.149 to 0.023)	−0.025	0.150
Food as a Reward or Punishment	0.130 (0.044 to 0.216)	0.052	0.00 3*
Variety of Food	0.050 (−0.036 to 0.136)	0.020	0.254
Liberal Attitude	−0.260 (−0.346 to −0.174)	−0.105	<0.001 *
Child’s Involvement	0.032 (−0.054 to 0.118)	0.013	0.469
Mealtime	0.116 (0.030 to 0.202)	0.047	0.008 *

BMI—body mass index; CI—confidence interval; * *p* < 0.05.

**Table 5:** Spearman correlation coefficients between factors of dietary behaviour and nutrition–related parenting practices among seven- to eight-year-old children.

	Unhealthy Food	Proteins	Plant-Based	Dairy	Confectionery
**Control of Unhealthy Food**	−0.255	0.011	0.054	−0.030	−0.028
***p***	<0.001 *	*0.554*	0.005 *	*0.120*	*0.152*
**Cost of and Preferences for Food**	0.072 *	−0.042	−0.278	0.055	−0.041
***p***	<0.001 *	0.030 *	<0.001 *	0.004 *	0.031 *
**Taking Care of Family Food**	−0.011	0.098	0.090	0.069	−0.108
***p***	*0.579*	<0.001 *	<0.001 *	<0.001 *	<0.001 *
**Encouragement**	−0.072	0.002	0.109	0.019	0.013
***p***	<0.001*	*0.924*	<0.001 *	*0.334*	*0.502*
**Pressure to Eat**	0.070	0.038	−0.060	0.035	0.049
***p***	<0.001 *	*0.050*	0.002 *	*0.065*	0.011 *
**Preparation of Food**	−0.124	0.015	0.217	0.051	−0.033
***p***	<0.001 *	*0.443*	<0.001 *	0.008 *	*0.087*
**Food as a Reward or Punishment**	0.157	0.002	−0.056	0.049	0.106
***p***	<0.001 *	*0.912*	0.003 *	0.012 *	<0.001 *
**Variety of Food**	0.037	0.222	0.324	0.060	−0.127
***p***	*0.052*	<0.001 *	<0.001 *	0.002 *	<0.001 *
**Liberal Attitude**	0.146	0.020	−0.090	0.042	0.078
***p***	<0.001 *	*0.298*	<0.001 *	0.031 *	<0.001 *
**Child’s Involvement**	0.063	0.015	0.090	0.075	−0.058
***p***	0.001 *	*0.451*	<0.001 *	<0.001 *	0.002 *
**Mealtime**	0.061	−0.043	−0.076	−0.008	0.007
***p***	0.001 *	0.025 *	<0.001 *	*0.676*	*0.731*

*n* = 2705; * *p* < 0.05.
